# Lifestyle and poststroke recovery: A 2-sample Mendelian randomization analysis

**DOI:** 10.1097/MD.0000000000045115

**Published:** 2025-10-10

**Authors:** Liqun Jiang, Huimin Ding, Hyun Seo, Buongo Chun

**Affiliations:** a Graduate School of Physical Education, Myongji University, Yongin, Republic of Korea; b Department of Sport and Leisure Studies, Graduate School, Korea University, Sejong, Republic of Korea.

**Keywords:** genetic epidemiology, lifestyle factors, Mendelian randomization, physical activity, stroke rehabilitation

## Abstract

Stroke is a leading cause of long-term disability and mortality worldwide. Modifiable lifestyle factors (including physical activity, diet, sleep, and psychological health) may influence poststroke recovery. However, evidence from observational studies is limited by confounding and reverse causation. We conducted a 2-sample Mendelian randomization (MR) analysis to investigate causal associations between 12 lifestyle-related exposures and stroke recovery outcomes. Genetic instruments (11–26 single nucleotide polymorphisms, all *F*-statistics > 10) were obtained from the MRC-IEU consortium (sample sizes 64,949–461,460), and outcome data were derived from the GISCOME consortium (6021 ischemic stroke patients, mean age ~65 years, predominantly middle-aged and older adults). Harmonization procedures ensured allele alignment, with palindromic and weak instruments removed. Causal estimates were obtained using inverse variance weighted (IVW), weighted median, MR-Egger, simple mode, and weighted mode methods. Sensitivity analyses included heterogeneity tests, MR-PRESSO, and leave-one-out analyses. Sedentary behavior was associated with poorer recovery (IVW odds ratio [OR] = 0.01, 95% confidence interval [CI]: 0.01–0.03, *P* < .05), whereas moderate-intensity physical activity showed beneficial associations (IVW OR = 4.91, 95% CI: 2.13–11.34, *P* = .01). Higher body mass index was negatively associated with recovery (IVW OR = 0.04, 95% CI: 0.01–0.09, *P* < .05). A low-calorie diet demonstrated significant benefit (IVW OR = 18.37, 95% CI: 1.05–3.20 × 10², *P* < .05). Smoking (IVW OR = 0.01, 95% CI: 0.01–0.09, *P* < .05), psychological distress (IVW OR = 0.01, 95% CI: 0.01–0.03, *P* < .05), and insomnia (IVW OR = 0.01, 95% CI: 0.01–0.02, *P* < .05) were negatively associated with recovery. No significant associations were observed for low- or high-intensity activity, alcohol abstinence, coffee intake, or household income. Sensitivity analyses indicated no heterogeneity or directional pleiotropy (MR-Egger intercept *P* > .05), and MR-PRESSO confirmed the robustness of causal estimates. This MR study provides genetic evidence that sedentary behavior, high body mass index, smoking, psychological distress, and insomnia may impair stroke recovery, whereas moderate-intensity physical activity and low-calorie diets may improve recovery. These findings emphasize the importance of integrating lifestyle modification into personalized stroke rehabilitation strategies.

## 1. Introduction

Stroke, also known as cerebrovascular accident, is an acute neurological dysfunction caused by either vascular obstruction or rupture, and is generally classified into 2 main types: ischemic and hemorrhagic stroke.^[1]^ With the accelerating global trend of population aging, both the incidence and mortality of stroke have risen significantly, making it one of the leading causes of death and long-term disability worldwide.^[2]^ Stroke not only results in substantial medical expenditures and prolonged caregiving burdens, but also frequently leaves patients with sequelae such as hemiplegia and aphasia. Inadequate rehabilitation interventions may exacerbate functional impairments and elevate the risk of recurrence.^[3]^ Therefore, improving patient outcomes and reducing the socioeconomic burden have become central concerns in both clinical practice and public health.

Evidence suggests that lifestyle interventions play a pivotal role in both the prevention and rehabilitation of stroke. By modifying behavioral habits, such interventions can mitigate vascular and neurological damage, thereby effectively controlling disease progression.^[4]^ A low-sodium, low-fat diet combined with adequate high-quality protein intake helps regulate blood pressure, lipid profiles, and body weight^[5,6]^; smoking cessation and alcohol restriction reduce endothelial injury and create a more favorable microenvironment for neural repair^[7]^; meanwhile, adequate sleep and psychological regulation alleviate stress responses and chronic inflammation, thereby promoting neural recovery and cerebral function reconstruction.^[8]^ Although current clinical practices have demonstrated the effectiveness of these strategies, their underlying mechanisms and the long-term sustainability of effects warrant further investigation in larger-scale studies.

Among various intervention strategies, exercise-based rehabilitation is considered the most promising core approach.^[9]^ Regular aerobic exercise enhances cardiopulmonary function, improves blood circulation, and reduces the risk of arteriosclerosis and stroke recurrence.^[10]^ For patients with hemiplegia, resistance training and balance exercises can improve muscle strength and joint mobility, thereby facilitating functional recovery. An individualized exercise prescription should be tailored to the severity of the condition, type of functional impairment, and psychological status, with progressive intensity adjustments to ensure both safety and therapeutic efficacy.^[9,11]^ Notably, the combined application of exercise interventions with psychological support, occupational therapy, and traditional modalities such as acupuncture can produce synergistic effects across physiological and psychological domains, thereby significantly enhancing overall rehabilitation outcomes.^[12]^

However, traditional observational studies are often subject to confounding and reverse causation, making it challenging to draw reliable inferences about the relationships between lifestyle factors and stroke rehabilitation outcomes. Mendelian randomization (MR) offers a complementary approach by using genetic variants as instrumental variables (IVs) to approximate the structure of randomized controlled trials, thereby reducing the influence of certain biases.^[13]^ This study aims to apply a 2-sample MR design to systematically explore the potential associations and biological mechanisms linking lifestyle-related factors (such as dietary patterns, physical activity intensity, and sleep quality) with poststroke recovery outcomes. The objective is to contribute to a more informed understanding that may support the development of personalized rehabilitation strategies and provide scientific insights relevant to public health planning. While the findings may help refine clinical decision-making and contribute to the ongoing shift from empirical approaches to more tailored rehabilitation frameworks, further research will be needed to validate and extend these observations.

## 2. Methods

### 2.1. Study design

**Figure 1.**
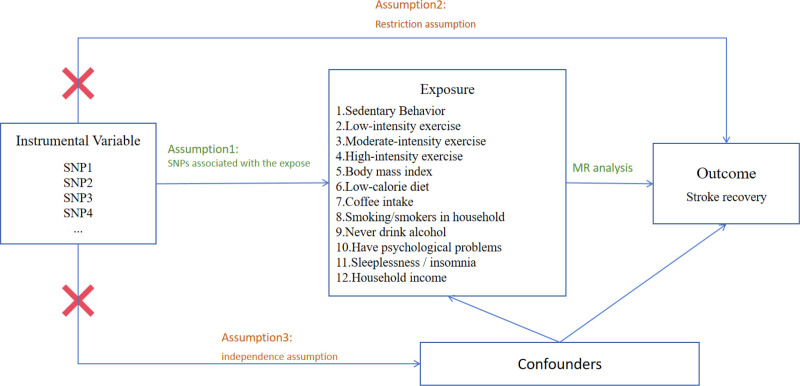
Conceptual framework of Mendelian randomization (MR) analysis.

To explore the causal relationships between multiple lifestyle factors and stroke rehabilitation outcomes, this study adopted a 2-sample MR approach, utilizing summary-level data from genome-wide association studies (GWAS) on both exposures and outcomes. Two-sample MR extracts genetic associations with exposures and outcomes from independent cohorts to estimate causal effects.^[14,15]^ To control for population stratification bias, the analysis was restricted to individuals of European ancestry.^[16]^ The validity of the MR analysis relies on 3 core assumptions: the relevance assumption, which requires that the IVs are strongly associated with the exposure (*P* < 5 × 10⁻⁸); the independence assumption, meaning that IVs are independent of potential confounders of the exposure–outcome relationship; and the exclusion restriction assumption, which states that IVs influence the outcome only through the exposure.^[17]^ This design mimics the framework of a randomized controlled trial by using genetic variants, such as single nucleotide polymorphisms (SNPs), to infer causal relationships. All analyses were conducted using publicly available GWAS data, which do not involve individual-level information and therefore do not require informed consent or ethical approval, in accordance with the STROBE-MR guideline.^[18]^ An overview of the MR study design is illustrated in Figure 1.

### 2.2. Data sources

All GWAS summary statistics for the exposure variables were obtained from the publicly accessible IEU OpenGWAS project (https://gwas.mrcieu.ac.uk/), which primarily includes participants aged 40 to 69 years at recruitment from the UK Biobank.^[19]^ Genetic data on poststroke functional recovery were derived from the GISCOME consortium, which integrates 12 studies conducted across the United States, Europe, and Australia, comprising a total of 6021 ischemic stroke patients with a mean age of approximately 65 years, representing predominantly middle-aged and older adults.^[20]^ To minimize population stratification bias, only individuals of European ancestry were included. Stroke recovery was assessed using the Modified Rankin Scale, a widely validated and commonly used measure of functional outcomes in stroke patients.^[21]^ Detailed GWAS dataset information is summarized in Table 1.

**Table 1:** GWAS data sources for MR analysis.

Trait	Consortium	Sample size	GWAS-ID/PMID
Sedentary behavior: time spent watching television (TV)	MRC-IEU	437,887	ukb-b-5192
Low-intensity exercise: types of physical activity in last 4 wk: walking for pleasure	MRC-IEU	460,376	ukb-b-7337
Moderate-intensity exercise: number of days/week of moderate physical activity 10 + min	MRC-IEU	440,266	ukb-b-4710
High-intensity exercise: number of days/week of vigorous physical activity 10 + min	MRC-IEU	440,512	ukb-b-151
BMI: body mass index	MRC-IEU	461,460	ukb-b-19953
Low-calorie diet: type of special diet followed: low calorie	MRC-IEU	64,949	ukb-b-15768
Coffee intake	MRC-IEU	428,860	ukb-b-5237
Smoking/smokers in household	MRC-IEU	311,142	ukb-a-18
Never drink alcohol: alcohol drinker status: never	MRC-IEU	336,965	ukb-a-226
Have psychological problems: seen a psychiatrist for nerves, anxiety, tension or depression	MRC-IEU	460,702	ukb-b-18336
Sleeplessness/insomnia	MRC-IEU	462,341	ukb-b-3957
Household income: average total household income before tax	MRC-IEU	397,751	ukb-b-7408
Stroke recovery: genome-wide single-variant associations for Modified Rankin Scale score 0–2 vs 3–6 adj stroke severity	GISCOME	6021	30796134

GWAS = genome-wide association studies, MR = Mendelian randomization.

### 2.3. Selection of IVs

To ensure the validity of IVs, SNPs were selected based on a genome-wide significance threshold of *P* < 5 × 10⁻⁸, followed by linkage disequilibrium clumping to ensure independence between SNPs (*r*² < 0.001 within a 10 Mb window).^[22]^ Weak instruments were excluded by calculating the *F*-statistic, retaining only SNPs with *F* > 10 to avoid weak instrument bias.^[23]^ To enhance the specificity of the analysis, SNPs directly associated with stroke recovery outcomes were identified and excluded using the LDtrait tool (https://ldlink.nih.gov/?tab=ldtrait), thereby reducing potential pleiotropic effects that might bias causal inference.

### 2.4. Harmonization of exposure and outcome datasets

Exposure and outcome GWAS datasets were harmonized to ensure consistent alignment of effect alleles across both sources. SNPs were matched by rsID, and effect alleles were aligned so that they corresponded to the same genetic variant in both datasets. Where necessary, effect estimates were flipped to maintain allele orientation consistency. Palindromic SNPs with ambiguous strand orientation (A/T or G/C with allele frequency close to 0.5) were excluded. Harmonization was performed using the harmonise_data() function in the TwoSampleMR R package (MRC Integrative Epidemiology Unit, University of Bristol, Bristol, UK).^[13]^

### 2.5. MR analysis

MR analyses were performed using 3 complementary statistical approaches: inverse variance weighted (IVW), weighted median (WM), and MR-Egger regression, to examine the potential relationships between selected exposures and stroke recovery outcomes. The IVW method estimates an overall effect by combining the Wald ratios of individual SNPs using inverse variance weights. The WM method conducts a weighted regression based on the median of SNP-specific estimates, offering greater robustness when a proportion of the instruments may be invalid. MR-Egger regression introduces an intercept term to detect and adjust for potential horizontal pleiotropy. Prior to the main analyses, MR-PRESSO was applied to identify and remove outlier SNPs, and the models were re-estimated accordingly to assess the consistency of the results. A *P*-value < .05 was considered statistically significant in all tests.^[24,25]^

### 2.6. Sensitivity analysis

To ensure the robustness of the MR results, several sensitivity analyses were conducted. Cochran Q test was used to assess heterogeneity among SNPs; when *P* < .05, a random-effects IVW model was applied. The MR-Egger intercept test was performed to detect horizontal pleiotropy, with *P* > .05 indicating the absence of directional pleiotropy. A leave-one-out analysis was also conducted to examine the influence of individual SNPs on the overall causal estimates. In addition, funnel plots were generated to visually assess potential horizontal pleiotropy rather than publication bias, consistent with MR methodological standards. Finally, MR-PRESSO was employed to detect and correct for outlier SNPs, thereby enhancing the validity and robustness of the causal inference.^[26–28]^

## 3. Results

### 3.1. Selection of IVs

After systematic screening, 12 lifestyle-related phenotypes were selected as IVs. These variables were associated with 24, 18, 26, 12, 20, 17, 23, 19, 11, 17, 22, and 19 SNPs, respectively, corresponding to different domains of behavioral and lifestyle exposures: sedentary behavior (time spent watching television), low-intensity exercise (walking for pleasure in the last 4 weeks), moderate-intensity exercise (number of days per week engaging in ≥ 10 minutes of moderate physical activity), high-intensity exercise (number of days per week engaging in ≥ 10 minutes of vigorous physical activity), body mass index (BMI), low-calorie diet (self-reported adherence to a low-calorie diet), coffee intake, smoking or smokers in the household, alcohol abstinence (never drank alcohol), mental health problems (history of seeing a psychiatrist for nerves, anxiety, tension, or depression), sleeplessness/insomnia, and household income (average total household income before tax). After harmonization and outlier removal, the final set of IVs was confirmed, and their validity was evaluated. The results showed that all selected instruments had *F*-statistics >10, indicating no evidence of weak instrument bias (Table 2).

**Table 2:** Summary of instrumental variables (SNPs) used in MR analyses.

Expose	SNP	chr.exposure	NEA	EA	EAF	Base	SE	*P*	*F*
Sedentary behavior	rs73078367	3	C	T	0.117	-0.019	0.002	5.6E-14	56.494
	rs73082337	3	C	G	0.120	-0.017	0.002	2.8E-12	48.802
	rs78807522	3	C	A	0.119	-0.017	0.002	3.7E-12	48.253
	rs6774721	3	G	A	0.140	-0.018	0.002	1.1E-14	59.640
	rs73088122	3	C	T	0.142	-0.018	0.002	1.1E-14	59.681
	rs73088161	3	C	T	0.139	-0.017	0.002	2.5E-13	53.564
	rs7638643	3	C	T	0.138	-0.017	0.002	5E-13	52.216
	rs73074830	3	A	C	0.142	-0.017	0.002	2.5E-13	53.597
	rs16891325	6	T	C	0.158	-0.016	0.002	1E-13	55.272
	rs74571768	6	G	A	0.158	-0.016	0.002	1E-13	55.321
	rs16891344	6	G	T	0.158	-0.016	0.002	8.6E-14	55.672
	rs6937215	6	T	C	0.629	0.012	0.002	3.2E-14	57.584
	rs6905544	6	A	G	0.602	0.012	0.002	9.7E-14	55.431
	rs9320823	6	T	C	0.603	0.012	0.002	1.2E-13	54.938
	rs4412208	6	C	T	0.559	0.011	0.002	2.1E-12	49.399
	rs9401452	6	T	C	0.393	-0.012	0.002	2.4E-13	53.641
	rs7760876	6	C	T	0.437	-0.011	0.002	3.4E-12	48.432
	rs12663336	6	C	A	0.437	-0.011	0.002	4E-12	48.141
	rs13194250	6	C	T	0.437	-0.011	0.002	3.5E-12	48.366
	rs7742854	6	C	T	0.392	-0.012	0.002	2.4E-13	53.667
	rs4458695	6	G	A	0.393	-0.012	0.002	2E-13	53.970
	rs901628	6	C	G	0.392	-0.012	0.002	2.1E-13	53.874
	rs34522021	9	C	T	0.454	-0.012	0.002	1.1E-13	55.140
	rs7029718	9	G	A	0.415	-0.013	0.002	1.1E-16	68.762
Low-intensity exercise	rs12089815	1	G	A	0.549	0.005	0.001	2.4E-08	31.129
	rs12042107	1	T	C	0.549	0.005	0.001	1.9E-08	31.623
	rs748211	2	T	C	0.611	-0.005	0.001	2E-08	31.506
	rs10203072	2	T	C	0.600	-0.005	0.001	1.7E-08	31.816
	rs10206338	2	G	A	0.577	-0.005	0.001	1E-08	32.812
	rs1866823	8	G	A	0.545	0.006	0.001	2.4E-09	35.596
	rs2679053	11	A	G	0.599	0.006	0.001	4E-09	34.630
	rs1023955	11	T	G	0.598	0.006	0.001	3.3E-09	34.975
	rs1023956	11	T	C	0.598	0.006	0.001	3.3E-09	34.994
	rs7957096	12	A	G	0.712	-0.006	0.001	4.3E-09	34.505
	rs1790121	12	G	A	0.710	-0.006	0.001	1.5E-08	32.006
	rs1611973	12	C	G	0.713	-0.006	0.001	2E-08	31.526
	rs1727312	12	G	T	0.713	-0.006	0.001	1.9E-08	31.588
	rs11610710	12	G	A	0.713	-0.006	0.001	3.5E-08	30.381
	rs1790134	12	T	C	0.713	-0.006	0.001	3.7E-08	30.281
	rs7196161	16	G	A	0.633	-0.006	0.001	2E-09	35.930
	rs889548	16	C	T	0.360	0.006	0.001	1.2E-09	36.893
	rs2855475	16	G	A	0.639	-0.006	0.001	4.7E-09	34.309
Moderate-intensity exercise	rs67092078	6	G	A	0.109	-0.051	0.008	1.6E-10	40.862
	rs55834529	6	A	G	0.109	-0.051	0.008	2.4E-10	40.105
	rs71537559	6	G	C	0.111	-0.050	0.008	2.2E-10	40.238
	rs66841633	6	G	T	0.111	-0.050	0.008	3.7E-10	39.244
	rs34150729	6	T	C	0.110	-0.050	0.008	3.2E-10	39.527
	rs13191227	6	G	C	0.110	-0.050	0.008	3.4E-10	39.427
	rs13195040	6	A	G	0.111	-0.049	0.008	4.5E-10	38.863
	rs35848276	6	C	T	0.112	-0.050	0.008	2.7E-10	39.890
	rs35715914	6	C	T	0.111	-0.051	0.008	2.6E-10	39.973
	rs35501037	6	T	A	0.116	-0.048	0.008	4.5E-10	38.871
	rs13195728	6	T	C	0.116	-0.049	0.008	3.5E-10	39.351
	rs200483	6	G	A	0.135	-0.048	0.007	2.7E-11	44.387
	rs71559050	6	C	A	0.116	-0.049	0.008	4E-10	39.134
	rs67101035	6	C	G	0.116	-0.049	0.008	3.8E-10	39.196
	rs13199772	6	A	G	0.115	-0.049	0.008	3.9E-10	39.155
	rs13199906	6	C	G	0.115	-0.049	0.008	3.6E-10	39.306
	rs13199649	6	C	T	0.113	-0.051	0.008	1.2E-10	41.399
	rs71537572	6	T	C	0.112	-0.051	0.008	1.1E-10	41.639
	rs71559067	6	A	T	0.113	-0.051	0.008	1E-10	41.786
	rs13200214	6	C	T	0.113	-0.051	0.008	1E-10	41.793
	rs71559070	6	G	A	0.113	-0.051	0.008	7.6E-11	42.364
	rs13203816	6	T	C	0.114	-0.051	0.008	8.6E-11	42.123
	rs13205911	6	C	T	0.113	-0.051	0.008	5.4E-11	43.019
	rs35098436	6	T	C	0.114	-0.051	0.008	7.3E-11	42.425
	rs13195291	6	G	A	0.114	-0.051	0.008	1E-10	41.753
	rs13197633	6	G	A	0.113	-0.051	0.008	7.6E-11	42.350
High-intensity exercise	rs4856569	3	A	G	0.618	-0.027	0.004	3.4E-10	39.433
	rs2033526	3	A	C	0.618	-0.026	0.004	4.9E-10	38.722
	rs3129837	6	A	G	0.175	-0.031	0.005	1.1E-08	32.685
	rs3132650	6	A	G	0.174	-0.031	0.005	9.3E-09	32.984
	rs10259097	7	T	C	0.291	0.028	0.005	5.8E-10	38.393
	rs28639844	7	A	T	0.322	0.026	0.004	3.5E-09	34.905
	rs10282424	7	G	A	0.320	0.027	0.004	8.2E-10	37.702
	rs28844277	7	C	T	0.373	0.026	0.004	3.1E-09	35.106
	rs17167199	7	C	T	0.440	0.025	0.004	1.7E-09	36.279
	rs12707131	7	A	G	0.392	-0.032	0.004	8.1E-14	55.792
	rs307676	9	A	G	0.377	-0.025	0.004	8.7E-09	33.105
	rs35748266	9	G	C	0.277	-0.025	0.005	4.6E-08	29.886
Body mass index	rs567359	2	A	G	0.400	-0.024	0.002	1.9E-33	145.284
	rs551573	2	G	A	0.400	-0.024	0.002	1.5E-33	145.737
	rs2918630	2	T	C	0.429	-0.026	0.002	6.1E-38	165.808
	rs475868	2	A	G	0.429	-0.026	0.002	7E-38	165.544
	rs11684464	2	A	G	0.401	-0.024	0.002	3.1E-32	139.676
	rs73052033	3	T	C	0.185	-0.030	0.003	7.7E-33	142.466
	rs57527860	3	T	G	0.185	-0.030	0.003	3.1E-32	139.727
	rs55835921	3	T	C	0.186	-0.030	0.003	1.2E-31	137.066
	rs9473924	6	G	T	0.284	0.026	0.002	2.4E-32	140.191
	rs9473932	6	G	A	0.284	0.026	0.002	3E-32	139.754
	rs9463656	6	A	G	0.284	0.026	0.002	1.7E-32	140.874
	rs7305229	12	C	T	0.452	0.025	0.002	4.2E-35	152.801
	rs3784707	15	A	C	0.206	-0.030	0.002	1.8E-33	145.318
	rs28626095	15	T	C	0.223	-0.029	0.002	2.9E-35	153.567
	rs2278076	15	G	A	0.207	-0.029	0.002	6.2E-33	142.893
	rs4776976	15	C	T	0.223	-0.030	0.002	1.5E-35	154.920
	rs1026737	15	A	C	0.223	-0.030	0.002	1.3E-35	155.122
	rs4776978	15	A	G	0.223	-0.030	0.002	1.2E-35	155.248
	rs922494	15	T	C	0.225	-0.029	0.002	2.2E-35	154.092
	rs28539889	15	T	A	0.207	-0.029	0.002	5.3E-33	143.213
Low-calorie diet	rs6782815	3	A	C	0.416	-0.008	0.002	1.1E-05	19.300
	rs62295523	4	T	C	0.255	0.009	0.002	6.1E-06	20.444
	rs79034679	4	T	G	0.052	-0.016	0.004	5E-05	16.438
	rs10074788	5	T	C	0.265	0.008	0.002	4.7E-05	16.578
	rs12256551	10	A	C	0.359	0.008	0.002	9.2E-06	19.674
	rs11012753	10	C	T	0.287	0.008	0.002	4.2E-05	16.763
	rs12803325	11	A	T	0.345	-0.008	0.002	2.4E-05	17.805
	rs34228131	11	C	T	0.344	-0.008	0.002	2.2E-05	17.987
	rs634688	12	G	A	0.332	0.008	0.002	8.1E-06	19.907
	rs73348836	14	C	T	0.167	-0.010	0.002	4.5E-05	16.668
	rs9939973	16	G	A	0.425	0.009	0.002	3.6E-07	25.891
	rs55872725	16	C	T	0.405	0.009	0.002	2.6E-07	26.497
	rs7193144	16	T	C	0.396	0.009	0.002	1.7E-06	22.879
	rs9933509	16	T	C	0.415	0.009	0.002	1.1E-06	23.776
	rs3751812	16	G	T	0.395	0.009	0.002	7.4E-07	24.505
	rs7190396	16	T	G	0.395	0.009	0.002	8.8E-07	24.183
	rs7185735	16	A	G	0.396	0.009	0.002	7.8E-07	24.406
Coffee intake	rs10127720	1	T	C	0.741	0.012	0.002	5.5E-11	42.993
	rs4255425	1	G	C	0.670	0.011	0.002	1.2E-10	41.515
	rs79262512	7	G	A	0.038	0.027	0.004	3.4E-10	39.417
	rs34829274	7	A	C	0.248	0.012	0.002	4.8E-11	43.256
	rs1059787	7	C	T	0.246	0.012	0.002	4.4E-11	43.431
	rs11772454	7	A	C	0.160	-0.020	0.002	1.1E-19	82.470
	rs71399778	15	G	C	0.218	0.019	0.002	1.3E-22	95.700
	rs351242	15	G	A	0.759	-0.018	0.002	3.9E-21	89.049
	rs12913041	15	A	G	0.218	0.019	0.002	1.3E-22	95.734
	rs751527	15	T	C	0.349	-0.015	0.002	9.4E-20	82.736
	rs12440952	15	A	G	0.349	-0.015	0.002	1.3E-19	82.066
	rs7174179	15	A	G	0.713	0.015	0.002	3.4E-17	71.086
	rs62005806	15	G	A	0.072	0.034	0.003	3.4E-28	121.235
	rs12900662	15	T	C	0.576	0.018	0.002	3.3E-29	125.886
	rs12917120	15	T	C	0.663	0.018	0.002	7.4E-27	115.133
	rs7163636	15	T	C	0.483	-0.018	0.002	1.9E-29	126.990
	rs7241535	18	T	G	0.269	0.013	0.002	2.7E-12	48.870
	rs953442	18	T	C	0.247	0.014	0.002	5.2E-14	56.635
	rs12969709	18	C	A	0.266	0.015	0.002	2.1E-16	67.492
	rs12970134	18	G	A	0.266	0.015	0.002	3E-16	66.788
	rs67194783	18	C	T	0.264	0.015	0.002	1E-15	64.363
	rs76369026	22	A	T	0.014	-0.045	0.007	5.5E-11	42.975
	rs77143344	22	A	G	0.014	-0.045	0.007	5.4E-11	43.044
Smoking/smokers in household	rs13031036	2	T	A	0.699	-0.005	0.001	2.25E-07	26.810
	rs10496680	2	A	T	0.699	-0.005	0.001	2.35E-07	26.725
	rs143443301	2	C	A	0.992	-0.029	0.005	2.35E-08	31.183
	rs4893879	2	G	A	0.847	0.006	0.001	2.01E-06	22.586
	rs13432838	2	G	A	0.849	0.006	0.001	4.83E-06	20.906
	rs4893880	2	G	T	0.850	0.006	0.001	3.38E-06	21.586
	rs17771213	2	G	A	0.850	0.006	0.001	3.39E-06	21.584
	rs10183460	2	T	C	0.850	0.006	0.001	4.09E-06	21.223
	rs4682562	3	A	T	0.299	-0.005	0.001	1.09E-06	23.766
	rs34033556	4	G	C	0.625	-0.004	0.001	1.36E-06	23.330
	rs6852508	4	A	T	0.614	-0.004	0.001	2.12E-06	22.486
	rs10003054	4	T	A	0.614	-0.004	0.001	2.49E-06	22.171
	rs35723165	4	G	C	0.619	-0.004	0.001	2.41E-06	22.237
	rs9884355	4	G	C	0.615	-0.004	0.001	3.81E-06	21.361
	rs4328866	4	G	C	0.614	-0.004	0.001	3.33E-06	21.619
	rs13106780	4	C	G	0.619	-0.004	0.001	4.11E-06	21.213
	rs11728711	4	A	T	0.618	-0.004	0.001	3.42E-06	21.567
	rs13143943	4	C	G	0.619	-0.004	0.001	3.9E-06	21.312
	rs9998816	4	G	C	0.382	0.004	0.001	4.04E-06	21.247
Never drink alcohol	rs1005942	2	A	C	0.773	0.002	0.001	4.93E-06	20.864
	rs11681722	2	C	T	0.783	0.002	0.001	2.64E-06	22.059
	rs62110850	2	C	T	0.783	0.002	0.001	2.65E-06	22.053
	rs114026228	4	T	C	0.993	-0.013	0.003	3.19E-06	21.699
	rs145452708	4	G	C	0.990	-0.014	0.002	1.59E-10	40.922
	rs138495951	4	G	A	0.990	-0.014	0.002	1.71E-10	40.770
	rs141973904	4	C	T	0.990	-0.014	0.002	1.69E-10	40.798
	rs9382128	6	C	T	0.796	-0.002	0.001	4.47E-06	21.054
	rs79184077	7	A	G	0.958	0.005	0.001	1.05E-06	23.829
	rs11066719	12	T	C	0.477	-0.002	0.000	2.83E-06	21.929
	rs12312551	12	A	G	0.477	-0.002	0.000	1.68E-06	22.933
Have psychological problems	rs156744	6	G	A	0.246	-0.005	0.001	3.1E-10	39.585
	rs9468274	6	G	T	0.246	-0.005	0.001	3.7E-10	39.280
	rs9393884	6	A	G	0.240	-0.005	0.001	9.2E-11	41.986
	rs4713148	6	G	A	0.239	-0.005	0.001	5.3E-11	43.047
	rs9348794	6	T	A	0.239	-0.005	0.001	5.3E-11	43.063
	rs17774663	6	G	A	0.239	-0.005	0.001	5.3E-11	43.075
	rs11552219	6	C	T	0.239	-0.005	0.001	5.3E-11	43.065
	rs9357065	6	T	C	0.239	-0.005	0.001	4.8E-11	43.248
	rs35227624	6	A	G	0.239	-0.005	0.001	5.3E-11	43.071
	rs9380061	6	T	C	0.239	-0.005	0.001	5.3E-11	43.054
	rs3173443	6	T	G	0.239	-0.005	0.001	5.3E-11	43.064
	rs9380062	6	G	A	0.239	-0.005	0.001	5.7E-11	42.936
	rs2188100	6	G	A	0.164	-0.007	0.001	1.3E-14	59.453
	rs3129837	6	A	G	0.175	-0.006	0.001	2E-13	53.996
	rs3129840	6	G	C	0.174	-0.006	0.001	2.4E-13	53.648
	rs805303	6	G	A	0.373	-0.004	0.001	1.1E-10	41.584
	rs707916	6	G	A	0.356	-0.004	0.001	1.4E-10	41.172
Sleeplessness/insomnia	rs11184946	1	C	T	0.415	0.010	0.002	2.2E-11	44.808
	rs6703664	1	G	A	0.416	0.010	0.002	3.1E-11	44.109
	rs4603157	1	G	A	0.412	0.010	0.002	5.1E-11	43.142
	rs11184955	1	A	C	0.413	0.010	0.002	1E-10	41.785
	rs10821128	9	T	C	0.331	-0.010	0.002	1.2E-10	41.403
	rs7869969	9	A	G	0.331	-0.010	0.002	8.8E-11	42.061
	rs4744240	9	C	T	0.330	-0.010	0.002	8.1E-11	42.231
	rs10821139	9	G	T	0.331	-0.010	0.002	7.7E-11	42.331
	rs77602510	10	G	A	0.078	-0.018	0.003	2.7E-10	39.882
	rs12411886	10	C	A	0.077	-0.018	0.003	2.7E-10	39.867
	rs11191475	10	C	T	0.077	-0.017	0.003	3.7E-10	39.239
	rs12219027	10	T	C	0.077	-0.017	0.003	3.7E-10	39.276
	rs943037	10	C	T	0.077	-0.017	0.003	4E-10	39.113
	rs59695806	17	T	C	0.294	0.011	0.002	1.1E-11	46.206
	rs35173750	17	G	T	0.279	0.011	0.002	2E-10	40.471
	rs2239923	17	C	T	0.293	0.011	0.002	5.4E-12	47.536
	rs1053739	17	G	A	0.348	0.010	0.002	3.9E-10	39.170
	rs12603813	17	T	C	0.253	0.012	0.002	5.7E-12	47.419
	rs11653796	17	A	G	0.266	0.013	0.002	3E-15	62.293
	rs71373536	17	G	A	0.254	0.014	0.002	1.6E-15	63.561
	rs12051846	17	A	G	0.254	0.014	0.002	1.4E-15	63.750
	rs76233987	17	G	A	0.257	0.013	0.002	5.6E-15	61.024
Household income	rs9871380	3	G	A	0.307	0.022	0.003	1.9E-14	58.637
	rs6446272	3	G	A	0.307	0.022	0.003	2.3E-14	58.275
	rs9841110	3	C	G	0.305	0.022	0.003	3.5E-14	57.439
	rs9827021	3	C	A	0.305	0.022	0.003	4.3E-14	57.049
	rs3926569	3	C	T	0.305	0.022	0.003	4.7E-14	56.855
	rs13062429	3	A	G	0.695	-0.022	0.003	4.3E-14	57.027
	rs71324979	3	T	C	0.309	0.021	0.003	8.5E-14	55.682
	rs11921590	3	T	C	0.301	0.023	0.003	3.8E-15	61.775
	rs1873625	3	C	A	0.301	0.023	0.003	3.8E-15	61.804
	rs2172252	3	A	T	0.293	0.023	0.003	4.8E-15	61.344
	rs9822268	3	G	A	0.293	0.023	0.003	5.5E-15	61.056
	rs2883059	3	T	C	0.425	-0.019	0.003	3.2E-13	53.085
	rs34034116	3	C	A	0.428	-0.019	0.003	1E-12	50.837
	rs62262093	3	T	C	0.515	-0.019	0.003	2.8E-13	53.349
	rs190947	5	T	C	0.471	0.018	0.003	4.9E-12	47.744
	rs702565	5	A	C	0.471	0.018	0.003	4E-12	48.124
	rs1986252	5	A	G	0.524	-0.020	0.003	5.2E-14	56.664
	rs112415880	17	G	A	0.224	-0.023	0.003	9.1E-13	51.023
	rs17585214	17	C	T	0.225	-0.023	0.003	6.1E-13	51.815

MR = Mendelian randomization, SNP = single nucleotide polymorphism.

### 3.2. Associations between multiple lifestyle factors and poststroke recovery outcomes

As shown in Table 3, this study utilized MR to evaluate the causal effects of 12 lifestyle factors on stroke recovery outcomes, with the number of instrumental SNPs ranging from 11 to 26 per exposure. Effect estimates varied across MR methods, though the WM and IVW approaches produced relatively consistent findings. Several exposures demonstrated statistically significant associations (*P* < .05).

**Table 3:** Associations between multiple lifestyle factors and poststroke recovery outcomes.

Outcome	Sample size	OR 95% CI	*P*-value
Sedentary behavior	24		
MR Egger		0.01 (0.01–21.49)	.19
Weighted median		0.01 (0.01–0.06)	<.05
Inverse variance weighted		0.01 (0.01–0.03)	<.05
Simple mode		0.01 (0.01–0.39)	<.05
Weighted mode		0.01 (0.01–0.39)	<.05
Low-intensity exercise	18		
MR Egger		0.01 (0.01–2.9e + 40)	.78
Weighted median		0.67 (0.01–95.13)	.87
Inverse variance weighted		0.69 (0.01–45.48)	.86
Simple mode		1.41 (0.01–7.4e + 3)	.93
Weighted mode		1.59 (0.01–8.2e + 3)	.91
Moderate-intensity exercise	26		
MR Egger		0.09 (0.01–1.9e + 17)	.91
Weighted median		4.88 (1.74–13.76)	<.05
Inverse variance weighted		4.91 (2.13–11.34)	<.05
Simple mode		4.94 (0.73–33.63)	.11
Weighted mode		4.91 (0.68–35.42	.12
High-intensity exercise	12		
MR Egger		0.68 (0.01–5.4e + 5)	.95
Weighted median		0.36 (0.08–1.59)	.17
Inverse variance weighted		0.38 (0.12–1.18)	.09
Simple mode		0.13 (0.01–1.62)	.14
Weighted mode		1.29 (0.12–14.15)	.83
Body mass index	20		
MR Egger		0.01 (0.01–4.2e + 2)	.41
Weighted median		0.04 (0.01–0.14)	<.05
Inverse variance weighted		0.04 (0.01–0.09)	<.05
Simple mode		0.05 (0.01–0.32)	<.05
Weighted mode		0.05 (0.01–0.33)	<.05
Low-calorie diet	17		
MR Egger		166.69 (0.01–3.9e + 13)	.71
Weighted median		22.87 (0.66–7.9e + 2)	.08
Inverse variance weighted		18.37 (1.05–3.2e + 2)	<.05
Simple mode		5.54 (0.02–1.3e + 3)	.55
Weighted mode		33.52 (0.11–1.1e + 4)	.25
Drink coffee	23		
MR Egger		0.86 (0.01–270.94)	.94
Weighted median		1.08 (0.17–6.88)	.93
Inverse variance weighted		0.85 (0.2–3.68)	.83
Simple mode		1.45 (0.07–29.93)	.81
Weighted mode		1.28 (0.08–19.44)	.86
Smoking	19		
MR Egger		0.01 (0.01–2.4e + 4)	.32
Weighted median		0.01 (0.01–0.67)	<.05
Inverse variance weighted		0.01 (0.01–0.09)	<.05
Simple mode		0.01 (0.01–105.55)	.26
Weighted mode		0.01 (0.01–76.12)	.24
Never drink alcohol	11		
MR Egger		0.52 (0.01–1.1e + 8)	.95
Weighted median		0.34 (0.01–8.9e + 5)	.87
Inverse variance weighted		0.19 (0.01–3.5e + 4)	.79
Simple mode		0.01 (0.01–5.9e + 6)	.56
Weighted mode		0.01 (0.01–1.1e + 3)	.65
Psychological problems	17		
MR Egger		0.01 (0.01–2.5e + 19)	.72
Weighted median		0.01 (0.01–0.21)	<.05
Inverse variance weighted		0.01 (0.01–0.03)	<.05
Simple mode		0.01 (0.01–32.45)	.18
Weighted mode		0.01 (0.01–27.9)	.17
Insomnia	22		
MR Egger		0.74 (0.01–1.4e + 4)	.95
Weighted median		0.01 (0.01–0.09)	<.05
Inverse variance weighted		0.01 (0.01–0.02)	<.05
Simple mode		0.01 (0.01–1.39)	.08
Weighted mode		0.01 (0.01–0.9)	.06
Household income	19		
MR Egger		22.19 (0.01–7.8e + 7)	.69
Weighted median		3.26 (0.77–13.86)	.11
Inverse variance weighted		1.75 (0.57–5.34)	.32
Simple mode		3.81 (0.32–44.95)	.31
Weighted mode		3.81 (0.36–40.79)	.28

For sedentary behavior, both the WM (odds ratio [OR] = 0.01, 95% confidence interval [CI]: 0.01–0.03, *P* < .05) and IVW (OR = 0.01, 95% CI: 0.01–0.03, *P* < .05) methods suggested a strong inverse association with functional recovery. In contrast, no significant associations were observed for low-intensity physical activity across any method (all *P* > .05). For moderate-intensity physical activity, the WM (OR = 4.88, 95% CI: 1.94–12.27, *P* = .01) and IVW (OR = 4.91, 95% CI: 2.13–11.34, *P* = .01) methods indicated a positive association with stroke recovery, while other methods did not yield statistically significant results. No significant associations were detected for high-intensity activity (all *P* > .05).

Regarding BMI, consistent negative associations were observed across the WM (OR = 0.05, 95% CI: 0.02–0.11, *P* < .05), IVW (OR = 0.04, 95% CI: 0.01–0.09, *P* < .05), and mode-based methods (OR = 0.04, 95% CI: 0.01–0.08, *P* < .05). A low-calorie diet showed a significant effect only in the IVW model (OR = 18.37, 95% CI: 1.05–3.20 × 10², *P* < .05), while no significant associations were identified for coffee intake, alcohol abstinence, or household income (all *P* > .05).

Smoking exposure was negatively associated with recovery in both the WM (OR = 0.01, 95% CI: 0.01–0.08, *P* < .05) and IVW (OR = 0.01, 95% CI: 0.01–0.09, *P* < .05) methods, although MR-Egger results were not significant. Similarly, psychological distress showed robust negative associations (IVW OR = 0.01, 95% CI: 0.01–0.03, *P* < .05; WM OR = 0.01, 95% CI: 0.01–0.02, *P* < .05). Insomnia was also significantly associated with poorer recovery outcomes in both the IVW (OR = 0.01, 95% CI: 0.01–0.02, *P* < .05) and WM (OR = 0.01, 95% CI: 0.01–0.02, *P* < .05) models.

### 3.3. Sensitivity analyses

As shown in Table 4, Cochran Q tests yielded *P*-values > .05 across all exposures in the sensitivity analyses, indicating no significant heterogeneity among the IVs. To account for potential variability, the random-effects IVW method was used as the primary analytical approach due to its robustness under moderate heterogeneity.

**Table 4:** Results of sensitivity analyses in Mendelian randomization.

Expose	Heterogeneity				Pleiotropy
	MR-Egger method		IVW method		MR-Egger intercept
	Cochran Q	*P*-value	Cochran Q	*P*-value	*P*-value
Sedentary behavior	2.134254	.89	2.20568	.95	.79
Low-intensity exercise	0.71869	.97	0.794008	.97	.78
Moderate-intensity exercise	0.25465	.95	0.288226	.93	.85
High-intensity exercise	4.241266	.93	4.248527	.96	.93
Body mass index	1.858038	.89	1.902161	.93	.83
Low-calorie diet	0.650427	.95	0.677972	.97	.87
Coffee intake	0.884463	.89	0.884465	.96	.91
Smoking/smokers in household	2.01826	.83	2.137381	.87	.73
Never drink alcohol	0.717435	.95	0.73521	.72	.89
Have psychological problems	0.139337	.94	0.140148	.79	.92
Sleeplessness/insomnia	3.000086	.92	4.366451	.94	.25
Household income	4.50155	.91	4.611092	.92	.74

**Figure 2.**
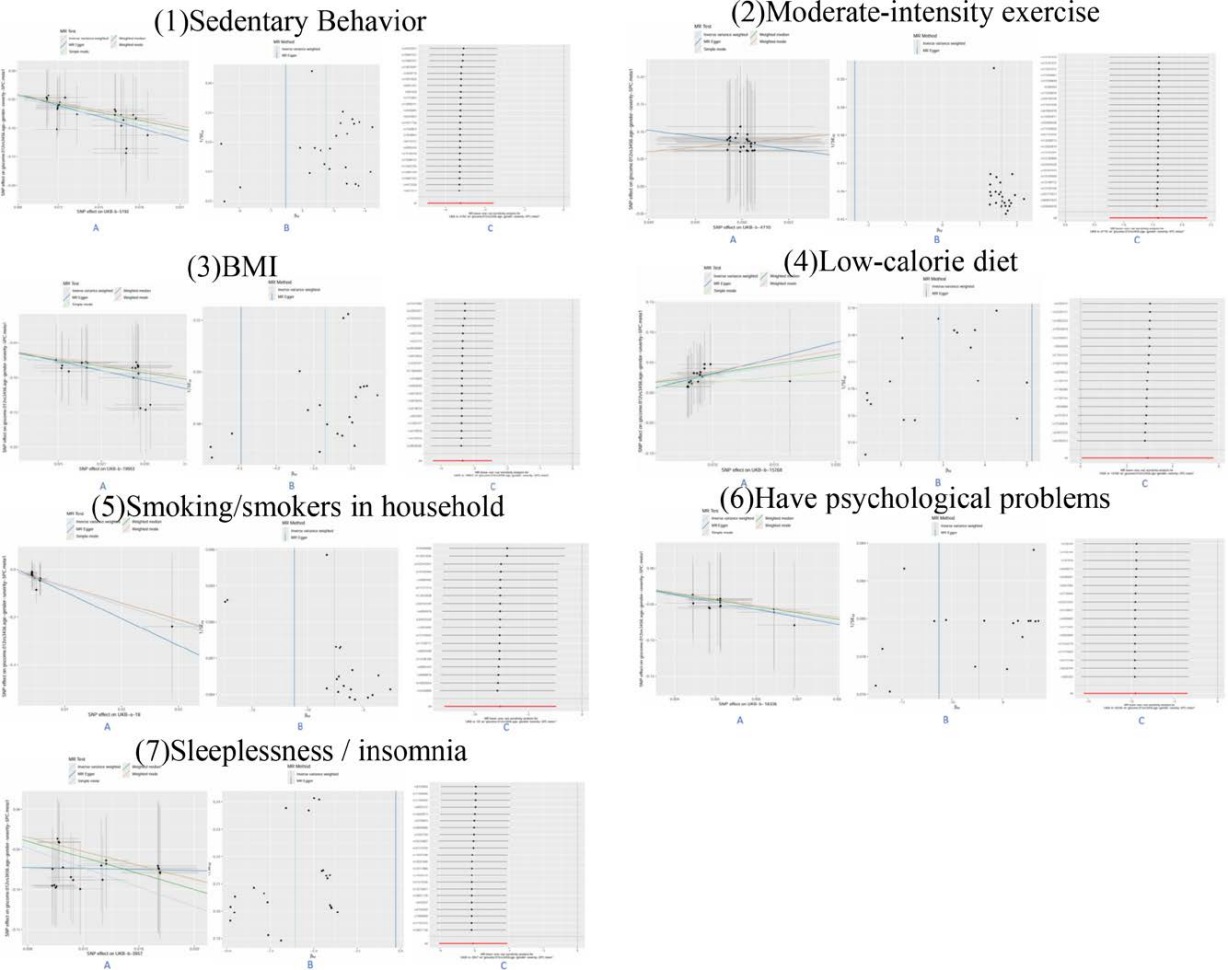
Sensitivity analyses of the Mendelian randomization (MR) results for lifestyle exposures on stroke recovery. Sensitivity analyses were performed for 7 exposures: (1) sedentary behavior, (2) moderate-intensity exercise, (3) body mass index (BMI), (4) low-calorie diet, (5) smoking or smokers in household, (6) psychological problems, and (7) sleeplessness/insomnia. For each exposure: (A) Scatter plots of SNP effects on the exposure versus stroke recovery, with regression lines estimated using different MR methods (inverse variance weighted, MR-Egger, weighted median, simple mode, and weighted mode). (B) Funnel plots of SNP estimates against their precision (1/SE). Vertical lines represent overall causal estimates from IVW and MR-Egger methods. Symmetry indicates the absence of substantial directional pleiotropy. (C) Leave-one-out analyses, where each point represents the MR estimate after excluding 1 SNP at a time. Consistent results across exclusions suggest robustness of the causal estimates. IVW = inverse variance weighted, SNP = single nucleotide polymorphism.

The MR-Egger intercept test and the corresponding scatter plot (Fig. 2A) showed *P*-values > .05, indicating no evidence of horizontal pleiotropy, despite a nonzero intercept that was not statistically significant. The funnel plot (Fig. 2B) demonstrated an approximately symmetric distribution, suggesting no apparent outliers or directional bias. In addition, the leave-one-out analysis (Fig. 2C) showed that the exclusion of any single SNP did not materially alter the overall estimates, supporting the robustness and stability of the results.

Furthermore, MR-PRESSO global and outlier tests were performed to further evaluate the influence of horizontal pleiotropy (Table 5). No significant outlier SNPs were detected across exposures, and the distortion tests showed no evidence of systematic pleiotropy. Importantly, the outlier-corrected estimates were nearly identical to the raw MR estimates, reinforcing the robustness of our findings.

**Table 5:** MR-PRESSO analysis results for lifestyle factors and stroke recovery.

Exposure	MR analysis	β	SE	Global *P*	Outlier SNPs	Distortion *P*	Change vs original
Sedentary behavior	Raw	-5.225	0.266	.92	0	–	No change
Sedentary behavior	Outlier-corrected	-5.225	0.266	–	–	–	-
Low-intensity exercise	Raw	-0.37	0.46	.87	0	–	No change
Low-intensity exercise	Outlier-corrected	-0.37	0.46	–	–	–	-
Moderate-intensity exercise	Raw	1.59	0.046	.77	0	–	No change
Moderate-intensity exercise	Outlier-corrected	1.59	0.046	–	–	–	-
High-intensity exercise	Raw	-0.97	0.36	.96	0	–	No change
High-intensity exercise	Outlier-corrected	-0.97	0.36	–	–	–	-
Body mass index	Raw	-3.34	0.14	.86	0	–	No change
Body mass index	Outlier-corrected	-3.34	0.14	–	–	–	-
Low-calorie diet	Raw	2.91	9.68	.69	0	–	No change
Low-calorie diet	Outlier-corrected	2.91	9.68	–	–	–	-
Drink coffee	Raw	-0.156	0.149	.95	0	–	No change
Drink coffee	Outlier-corrected	-0.156	0.149	–	–	–	-
Smoking	Raw	-5.75	0.91	.42	0	–	No change
Smoking	Outlier-corrected	-5.75	0.91	–	–	–	-
Never drink alcohol	Raw	-1.65	1.68	.49	0	–	No change
Never drink alcohol	Outlier-corrected	-1.65	1.68	–	–	–	-
Psychological problems	Raw	-9.469	0.286	.88	0	–	No change
Psychological problems	Outlier-corrected	-9.469	0.286	–	–	–	–
Insomnia	Raw	-6.07	0.46	.89	0	–	No change
Insomnia	Outlier-corrected	-6.07	0.46	–	–	–	-
Household income	Raw	0.56	0.287	.93	0	–	No change
Household income	Outlier-corrected	0.56	0.287	–	–	–	–

MR = Mendelian randomization, SNP = single nucleotide polymorphism.

## 4. Discussion

The findings of this study suggest that sedentary behavior may be associated with poorer stroke recovery outcomes, while moderate-intensity physical activity appears to be linked to more favorable functional outcomes. In contrast, low- and high-intensity physical activity did not show statistically significant associations in the present analysis. Prior studies have reported that prolonged sedentary behavior is associated with adverse neurological prognosis, and longer sitting time has been linked to increased metabolic risk, reduced cardiopulmonary function, and heightened inflammatory responses: all of which may negatively influence neural recovery processes. A recent systematic review also indicated that sedentary behavior may be associated with an elevated risk of stroke recurrence and related complications.^[29]^ In line with this, moderate-intensity physical activity (when performed regularly) has been proposed to support recovery by improving systemic circulation, facilitating cerebral collateral vessel development, and potentially promoting neuroplasticity and a more favorable metabolic environment.^[30,31]^ Exercise training has also been associated with increased secretion of neurotrophic factors in the brain,^[30]^ and moderate exercise is thought to have beneficial effects on immune function, inflammation regulation, and cardiovascular health.^[9]^ From a psychological perspective, moderate activity may help alleviate anxiety and depressive symptoms, which could indirectly support rehabilitation progress. Taken together, these findings (though not definitive) may suggest that the negative impact of sedentary behavior on stroke recovery could be related to reduced cerebral perfusion and limited activation of neuroplastic mechanisms.^[32]^ The lack of observable benefits from low-intensity activity may be due to insufficient physiological stimulation, while high-intensity activity, although potentially beneficial in certain populations, might exceed the optimal threshold for some stroke survivors, possibly leading to fatigue or cardiovascular strain. This could help explain the absence of significant effects in either direction for these activity levels.

Higher levels of BMI were found to be potentially associated with less favorable stroke recovery outcomes, whereas low-calorie dietary patterns appeared to be associated with more favorable recovery profiles. Previous epidemiological studies have suggested that elevated BMI is linked to increased risk of cardiovascular events, impaired glucose metabolism, dyslipidemia, and chronic inflammation: all of which may contribute to cerebrovascular burden and hinder neurological recovery. In clinical settings, higher BMI is often accompanied by decreased physical capacity, a greater number of comorbidities, and lower adherence to rehabilitation protocols, which may collectively interfere with recovery progress.^[33]^ As for low-calorie diets, prior nutrition intervention trials in both stroke prevention and rehabilitation contexts have reported their potential benefits. Mechanistically, caloric restriction has been associated with improved weight management and insulin sensitivity, along with more favorable lipid profiles and reduced inflammatory markers, thereby potentially creating a metabolic environment more conducive to neurorepair and plasticity.^[34]^ From nutritional and metabolic perspectives, low-calorie diets may help reduce metabolic overload and alleviate stress on the cardiovascular and nervous systems. Additionally, from immunological and neurobiological viewpoints, moderate energy restriction has been proposed to activate cellular signaling pathways associated with neuroprotection and tissue repair.^[35]^ Given these findings, integrating weight management and dietary optimization into individualized rehabilitation plans may be an important consideration in promoting functional recovery after stroke, although further research is needed to clarify the strength and consistency of these associations.^[36]^

Psychological conditions, such as depression and anxiety, along with insomnia, may be associated with less favorable stroke rehabilitation outcomes. Large-scale prospective studies have suggested that poststroke depression can reduce treatment adherence and may suppress neuroplasticity by downregulating neurotrophic factors and upregulating pro-inflammatory cytokines.^[37,38]^ Similarly, anxiety and other negative emotional states may lead to persistent sympathetic nervous system overactivation, resulting in increased vascular constriction and blood pressure fluctuations, which are detrimental to cerebral perfusion and tissue repair.^[39]^ Insomnia may exert comparable effects. On one hand, insufficient sleep can compromise the body’s regenerative capacity, disturb hormonal and neurotransmitter balance, and impair both cognitive and motor recovery efficiency.^[40]^ On the other hand, poor sleep quality may further exacerbate anxiety and depressive symptoms, potentially creating a vicious cycle.^[8]^ From the interdisciplinary perspectives of psychiatry, neuroscience, and sleep medicine, psychological distress and sleep disorders may share common mechanisms that hinder neurological recovery. These mechanisms include neurochemical imbalances driven by depression and anxiety, as well as disruption of cognitive and emotional regulatory circuits due to sleep disturbances.^[37,38,40]^ Given these observations, the early identification and management of psychological conditions and sleep disturbances in stroke survivors may play an important role in supporting optimal recovery, although further research is warranted to better understand these complex interactions.

Smoking may represent an important risk factor for poorer stroke rehabilitation outcomes, whereas no statistically significant associations were observed in this study for alcohol consumption, coffee intake, or household income. A substantial body of epidemiological and experimental research has demonstrated that nicotine and other harmful compounds in tobacco can impair endothelial function, increase the risk of arteriosclerosis and thrombosis, and potentially exacerbate cerebrovascular pathology, particularly in individuals recovering from stroke.^[41]^ Smoking has also been linked to reduced tolerance to rehabilitation exercises and lower oxygen saturation levels, which may collectively diminish rehabilitation efficiency. The lack of significant associations for alcohol, coffee, and income-related variables could be partly attributable to sample characteristics, individual variability, or differences in consumption patterns. For instance, moderate alcohol intake or coffee consumption may not exert substantial effects on recovery, especially in the absence of heavy or prolonged exposure.^[42]^ Previous studies on coffee intake have also yielded inconsistent results: while some report potential benefits for alertness and mood, others suggest that caffeine sensitivity may lead to adverse cardiovascular or sleep-related effects in certain individuals.^[43]^ Regarding household income, the absence of a significant association with stroke recovery may reflect context-specific factors, such as the availability of universal health coverage or public rehabilitation services. As noted in research from social medicine and health economics, socioeconomic status often influences access to medical care and quality of rehabilitation. However, in settings where basic healthcare infrastructure and social support systems are in place, the impact of income disparities on recovery outcomes may be attenuated.^[44]^ Taken together, the results suggest that smoking may directly affect both vascular and neurological repair mechanisms, which could explain its more pronounced negative association with recovery outcomes in this study. In contrast, the effects of alcohol, coffee, and income appear to be more complex and context-dependent, potentially influenced by dosage, individual physiology, and broader social determinants.

### 4.1. Strengths and limitations

This study applied MR to investigate potential associations between lifestyle factors (particularly physical activity) and stroke recovery. By using genetic variants as IVs, MR reduces the influence of confounding and reverse causation commonly seen in observational studies. The use of large-scale GWAS summary data from European populations enhanced statistical power and reduced population stratification bias. In addition, multiple sensitivity analyses (including MR-Egger, WM, and leave-one-out methods) were conducted to test the robustness of the findings. The inclusion of various physical activity intensities and additional lifestyle traits also allowed for a broader perspective on behavioral influences in poststroke outcomes.

However, several limitations should be acknowledged. The findings are based solely on European ancestry data, which may limit generalizability to other populations. The use of summary-level GWAS data prevented subgroup analyses by age, sex, or comorbidities, and nonlinear or interaction effects could not be explored. Statistical power may have been limited for exposures with small effect sizes or fewer instrumental SNPs, potentially contributing to null findings for some variables. Finally, gene–environment interactions and contextual factors such as socioeconomic status or healthcare access were not addressed and should be considered in future research.

## 5. Conclusion

This study suggests that sedentary behavior, elevated BMI, psychological distress, insomnia, and smoking may be associated with less favorable stroke recovery outcomes, whereas moderate-intensity physical activity and low-calorie diets appear to be linked with improved recovery. No significant associations were found for low- or high-intensity exercise, alcohol consumption, coffee intake, or household income, indicating that these factors may be influenced by contextual or individual-level differences. These findings support the value of personalized rehabilitation strategies that integrate physical activity, nutritional guidance, and psychological support, potentially enhanced by digital health tools in home-based settings. Further research involving diverse populations and incorporating multi-omics and phenotypic data is needed to clarify underlying mechanisms and inform more precise, effective interventions.

## Author contributions

**Conceptualization:** Liqun Jiang, Buongo Chun.

**Data curation:** Liqun Jiang, Hyun Seo.

**Formal analysis:** Liqun Jiang, Huimin Ding, Hyun Seo.

**Investigation:** Huimin Ding.

**Methodology:** Liqun Jiang, Huimin Ding, Hyun Seo.

**Project administration:** Buongo Chun.

**Supervision:** Buongo Chun.

**Visualization:** Liqun Jiang.

**Writing – original draft:** Liqun Jiang.

**Writing – review & editing:** Huimin Ding, Buongo Chun.
